# Addressing Pediatric Mental Health During COVID-19 and Other Disasters: A National Tabletop Exercise

**DOI:** 10.1017/dmp.2021.122

**Published:** 2021-04-19

**Authors:** Saloni Gupta, Merritt Schreiber, Tona McGuire, Christopher Newton

**Affiliations:** 1 David Geffen School of Medicine at UCLA, Los Angeles, CA, USA; 2 Department of Pediatrics, Lundquist Institute at Harbor – UCLA Medical Center, David Geffen School of Medicine at UCLA, Torrance, CA, USA; 3 Washington State Department of Health, Tumwater, WA, USA; 4 UCSF Benioff Children’s Hospital Oakland, CA, USA

**Keywords:** COVID-19, disaster response, mental health triage, PsySTART, tabletop exercise

## Abstract

**Objective:**

In the wake of the severe acute respiratory syndrome coronavirus 2 (SARS-CoV-2) pandemic, rapid identification of pediatric mental health risk is extremely important. The Western Regional Alliance for Pediatric Emergency Management held an integrated, interdisciplinary national tabletop exercise to familiarize mental health and non-mental health professionals with Psychological Simple Triage and Rapid Treatment (PsySTART), an evidence-based triage and incident management system used to evaluate new mental health risk impacts following exposure to traumatic events, such as coronavirus disease (COVID-19).

**Methods:**

Participants Participants were exposed to 3 practice cases that reflected a combination of “all hazards” scenarios and were asked to triage each case using PsySTART. Participants were asked to interpret results at both an individual site and aggregate county and/or state level.

**Results:**

The exercise had a total of 115 participants with a total of 156 discrete triage encounters. A user-defined operating picture was created with graphs of aggregate mental health risk data, generating cross-regional, real-time situational awareness. After the exercise, a vast majority of the participants reported confidence in their ability to use PsySTART in their practices.

**Conclusions:**

Participants are now better equipped with tools to perform mental health triage for early intervention during COVID-19 and other disasters and understand risk on a population level.

## Introduction

The importance of improved disaster planning for children in various disasters, including natural events such as earthquakes, hurricanes, and public health events, for example, the coronavirus disease (COVID-19) pandemic, as well as man-made events such as chemical, biological radiological or nuclear exposure, terrorism, and motor vehicle accidents is well known.^1^ National data show that 1 in 7 children in the United States has experienced a disaster in her or his lifetime.^2^ Because children have underdeveloped coping skills compared with adults, they may be susceptible to more severe psychological impacts of disasters.^3^ These effects fall on a continuum, ranging from short-term distress on a trajectory toward a resiliency pathway, to new-incidence disorders, such as posttraumatic stress disorder (PTSD).^4^ Children require specialized care to address these psychological stressors to not only prevent new-incidence disorders and worsening of pre-existing disorders, but also prevent the hindrance of growth, development, and school performance that can result from disaster-induced traumatic stress.^3^ Unfortunately, specialized pediatric mental health care is a relatively scarce commodity that is not available in large numbers or in every community. Significant progress has been made in understanding the continuum of impact since the Oklahoma City bombings, terrorism attacks of September 11, 2001, and Hurricane Katrina. However, pediatric mental health disaster planning remains incomplete, and the significant differences in disaster planning between pediatric and adult populations generally are not recognized.^3,5^

In the wake of the severe acute respiratory syndrome coronavirus 2 (SARS-CoV-2) pandemic and its clinical manifestation, COVID-19, it is important to identify children who have experienced trauma associated with COVID-19. This can include traumatic loss; life-threatening illness in loved ones, peers, or extended families; economic distress; interruptions in education; and loss of social support systems. Rapid identification provides an opportunity to intervene early. Psychological Simple Triage and Rapid Treatment (PsySTART) is a rapid, evidence-based triage and incident management system used to evaluate new mental health risk impacts following acute traumatic events at the individual and population level. The triage does not require direct interview of the child and can be done simply via gathering information on the child’s exposure to certain traumatic events.

In addition to identifying at-risk children, PsySTART can develop a user-defined operating picture (UDOP) of pediatric population-level mental health impact, leveraging key disaster systems of care, including hospitals and clinics, schools, and disaster relief settings. This UDOP aggregates individual-level triage data and generates a population-level impact of disasters. Aggregated data, including severity of and types of risk, can be shared across sites, counties, and state lines to promote real time, actionable intelligence using evidence-based situational awareness risk metrics. This may inform incident action plans (IAP) at the local, state, or national level. PsySTART uses a “floating algorithm,” which scales triage to population level impact and resources and has the potential to ethically inform the allocation of limited treatment resources in accordance with crisis standards of care protocols.^6^

Recently, The US Department of Health and Human Services Assistant Secretary for Preparedness and Response (HHS ASPR) created the Pediatric Disaster Centers of Excellence (PDCOE) Initiative to improve disaster response to pediatric populations.^7^ One component of the HHS ASPR PDCOE is an exercise and training element. This element is specific to pediatric populations and is designed to conform to the US Department of Homeland Security Exercise and Evaluation Program (HSEEP). Tabletop exercises (TTX) have shown promise in promoting disaster preparedness and enhancing health care professional core competencies toward improved care.^8^ Although TTX are common, there have been very few national level exercises that address or target the pediatric population and even fewer focused on population mental health response to children in mass casualty events (in fact, we are aware of none in the United States to date). Thus, in June and July of 2020, the Western Regional Alliance for Pediatric Emergency Management (WRAP-EM), funded by HHS ASPR, hosted a pediatric mental health focused TTX in order to familiarize leaders and frontline workers in pediatric health across the United States with the use and benefits of PsySTART in pediatric disaster planning, response, and recovery. The main objectives of this TTX were to use an integrated, interdisciplinary model to train participants across medical and mental health domains on the use of PsySTART triage systems and provide participants with exposure to the PsySTART capability to generate cross-regional and real-time situational awareness on pediatric mental health impact. Given this TTX took place in the midst of COVID-19, the training was structured so that following the TTX, facilities could immediately use PsySTART during this pandemic.

## Methods

A snowball sampling scheme was used to recruit participants across the United States, starting with participants from 2 groups funded by the US Department of Health and Human Services: WRAP-EM and the Eastern Great Lakes Pediatric Consortium for Disaster Response. Members of these 2 groups were asked to identify key stakeholders and frontline personnel across the United States who would benefit from the exercise.

To improve training transfer to diverse community providers, pediatric disaster case scenario video clips were chosen from public media and news sources that represented a variety of PsySTART risk factors applied to children in real-world, multi-hazard incidents. The PsySTART risk factors for this training were adapted to include additional risk factors related to COVID-19, including but not limited to confirmed exposure or infection with COVID-19, traumatic loss of loved or close friends, and family member separation due to COVID-19 risk or exposure. The scenarios reflected a combination of “all hazards” incidents, including a gunshot event, a flooding, a motor vehicle accident, improvised explosive device terrorism, and the interview of a child of a COVID-19 provider based on previous research on the impact of children of frontline medical providers.^9^

The TTX consisted of 5 identical instructor-led, 1-hour sessions held virtually. The first portion of the exercise consisted of training on the importance of early intervention in pediatric mental health after disasters. The second part of the exercise consisted of 3 practice scenarios. Participants viewed the audio and video clip scenarios and were then given time to individually triage each sample patient. The instructor reviewed the answers, and participants were given time to ask questions. At the conclusion, a UDOP was generated and shared with participants. The UDOP capability allowed participants to visualize, explore, and share data in the operational environment, thus allowing decision-makers to support timely action.

Participants were sent a post-exercise survey immediately after the exercise to evaluate usefulness of and satisfaction with the exercise and confidence in using PsySTART.

## Results

There was a total of 113 participants across 11 US states and 2 international participants. Participants spanned a range of roles, including physicians in pediatric emergency medicine, pediatric intensive care, child and general psychiatry, and general and behavioral pediatrics; psychologists and other mental health providers; and school crisis personnel. Participating sites included health care facilities, behavioral and public health departments, school districts, and disaster response teams. No individually identifiable information was collected, contained in the training or exercise, or used in analysis, and there was no evaluation of individual triage decisions in this training exercise.

**Figure 1.**
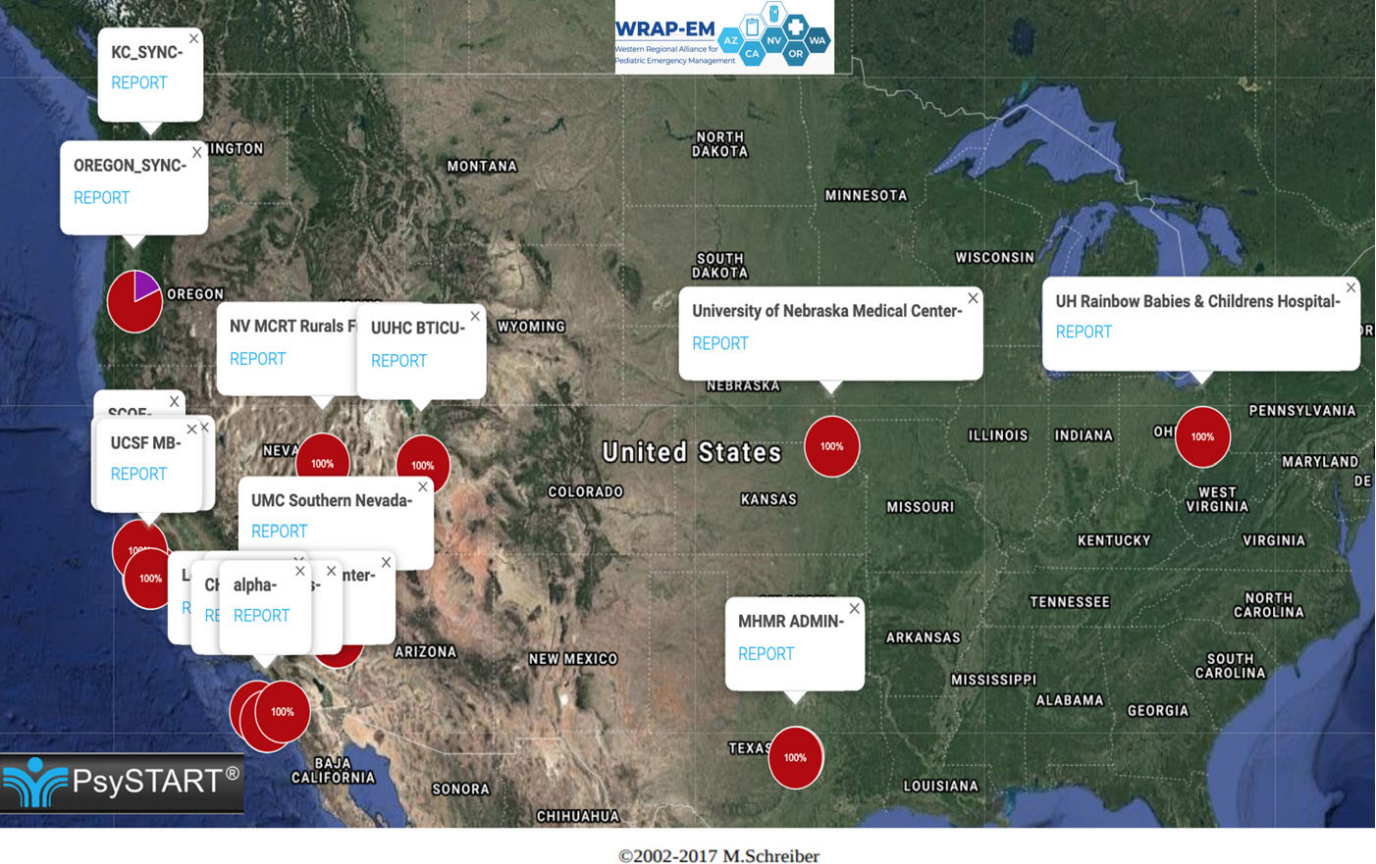
User-defined operating picture of tabletop exercise triage encounters within the United States.

**Figure 2.**
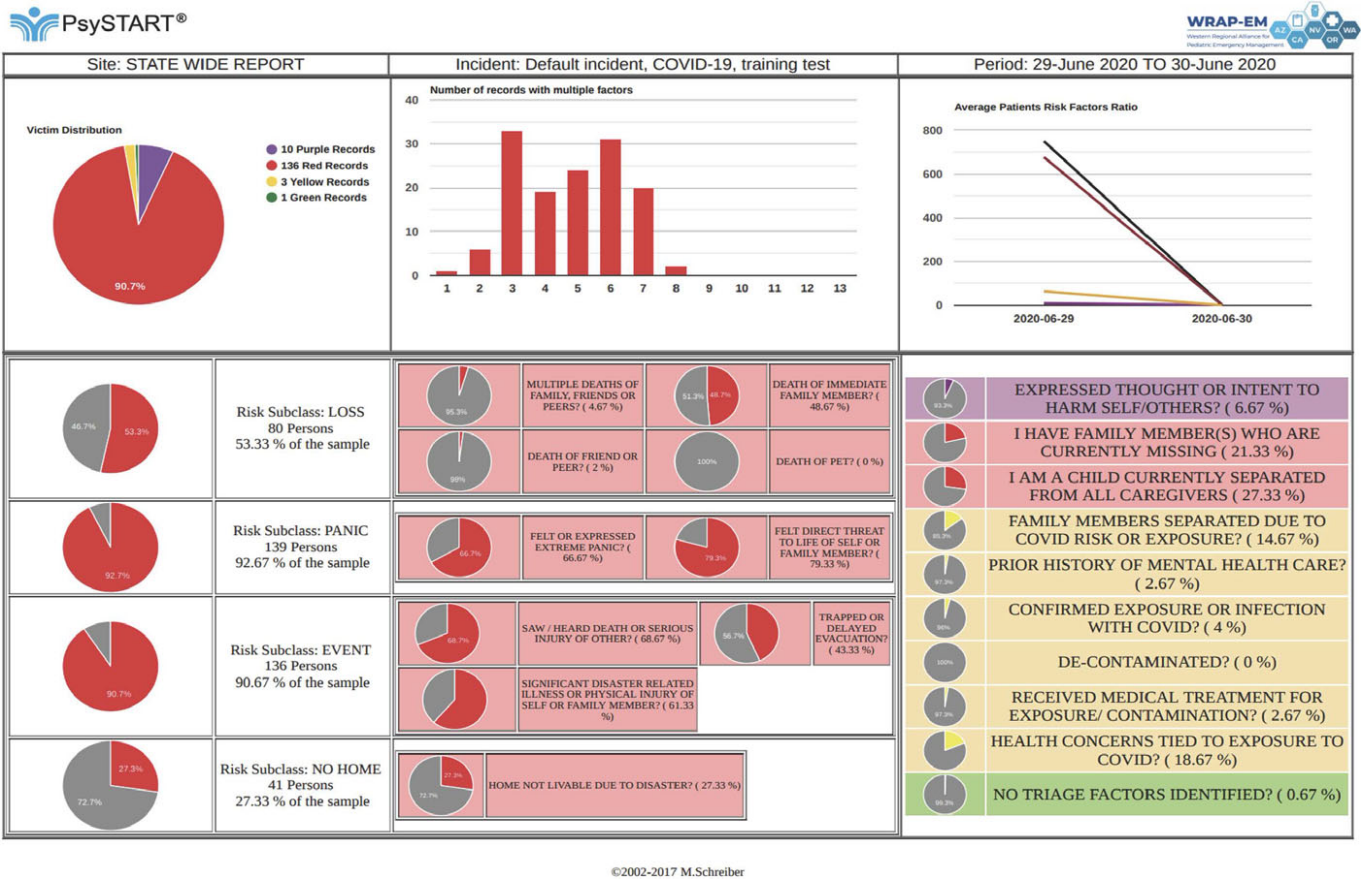
Aggregate risk data across all 156 triage encounters during the tabletop exercise.

PsySTART uses a color-coding system to delineate the presumptive individual risk, including purple for immediate danger and red for the presence of 1 or more evidence-based risk factors for either clinical PTSD or depression, and other incident/event specific indicators, such as unaccompanied children or children with pre-existing health issues. During the exercise, a total of 156 discrete triage encounters was entered into the PsySTART online system. The UDOP generated from these encounters showed the color-coded risk data unique to each participating site, as well as graphs of aggregate data within each state and/or county (Figure 1). Unlike a common operating picture, this UDOP functionality would allow the user to filter which information would be included or excluded from the data set (eg, a specific time frame or event). Aggregate risk data were also compiled and shared with participants (Figure 2).

Out of 115 participants, 67 (58%) completed the post-exercise survey. All respondents except 3 felt confident or very confident in their ability to use PsySTART moving forward in their own practices, with an average score of 4.40 on a scale of 1–5. All respondents except 4 also felt that this TTX helped them gain a better understanding of the potential benefits of using PsySTART in improving integrated care for children in disasters, with an average score of 4.57. All respondents except 2 were satisfied with the overall format of the exercise, with an average score of 4.52. All participants except 6 believed the exercise went into the appropriate amount of detail, with an average score of 4.49. In the free response section of the survey, participants expressed a desire to have longer duration exercises moving forward, with an increased number of scenarios, more time for individual triaging, and more time for group discussion before reviewing answers. Participants also expressed an interest in additional TTX covering the PsySTART reporting capabilities, which would allow users to explore aggregated local or statewide triage data following an acute traumatic event.

## Discussion

This TTX was established with the goals of training mental health and non-mental health providers on psychological risk detection through PsySTART to enhance situational awareness of pediatric disaster care capabilities after traumatic events and use this information to inform evidence-driven follow-up care, with a focus on the impacts of the COVID-19 pandemic on mental health. According to post-exercise survey responses, this TTX, the first to train on PsySTART specifically, also helped the majority of participants establish confidence in using PsySTART moving forward and helped them understand the role of PsySTART in improving integrated disaster care for children.

With the current COVID-19 pandemic and children being more likely to experience depression and anxiety due to isolation, this exercise was successful in providing a timely training to participants who work closely with children.^10^ The confidence and knowledge participants felt regarding the use of PsySTART will hopefully lead to not only a more timely identification of children who have experienced mental health risk factors of the COVID-19 pandemic and other disasters, but also be able to accurately recommend them for early linkage to mental health support. Moreover, participants are now equipped with a tool that allows them to rationally align limited resources to those with greater need, if the necessity arises, and to do so on a population-based level via the generation of a UDOP. Because the majority of participants reported that the TTX format worked well for them, virtual TTX could be effective strategies in improving disaster response across the United States, in general. The virtual nature of the TTX can serve as a template for similar TTX-type training needs for specific tools, allowing for participation across a large geographic area in real time.

## Limitations

Although cases were triaged into the PsySTART system during the exercise, the triage encounters were not mapped to the cases. For this reason, the end user accuracy of the triage encounters was not measurable, and thus it was not possible to quantitatively measure the effectiveness of the exercise or identify gaps to address in future exercises. We have been actively considering doing follow-up TTX to address this gap. This was a TTX with a limited and self-selected group of participants and not a full simulation.

## Conclusions

The WRAP-EM introduction to PsySTART TTX was successful in its efforts to demonstrate situational awareness across multiple US states. Participants’ knowledge of early mental health intervention and confidence in using PsySTART during the COVID-19 pandemic and beyond increased following the exercise. Pediatric leaders and frontline workers, nationally, are now better equipped with tools to identify risk level, to understand population-level impact, and to use aggregate data to ethically allocate resources appropriately.
